# A Scoping Review: Social Participation as a Cornerstone of Successful Aging in Place among Rural Older Adults

**DOI:** 10.3390/geriatrics3040075

**Published:** 2018-10-29

**Authors:** Lisa F. Carver, Rob Beamish, Susan P. Phillips, Michelle Villeneuve

**Affiliations:** 1 Department of Sociology, Faculty of Arts and Science, Queen’s University, Kingston, ON K7L 3N6, Canada; rob.beamish@queensu.ca; 2 Department of Family Medicine, School of Medicine, Queen’s University, Kingston, ON K7L 5N6, Canada; susan.phillips@dfm.queensu.ca; 3 Centre for Disability Research and Policy, Faculty of Health Sciences, The University of Sydney, Lidcombe, NSW 2141, Australia; michelle.villeneuve@sydney.edu.au

**Keywords:** rural, older adults, human–animal bond, social participation, aging in place

## Abstract

Despite obstacles, many rural-dwelling older adults report that positive aspects of rural residence, such as attachment to community, social participation, and familiarity, create a sense of belonging that far outweighs the negative. By being part of a community where they are known and they know people, rural elders continue to find meaning, the key to achieving successful aging in this last stage of life. This scoping review explored factors influencing social participation and, through it, successful aging among rural-dwelling older adults. We sought to answer the question: what factors enhance or detract from the ability of rural-dwelling older adults to engage in social participation in rural communities? The scoping review resulted in 19 articles that highlight the importance of supports to enable older people to spend time with others, including their pets, engage in volunteer and community activities, and help maintain their home and care for their pets. Overall, the lack of services, including local health care facilities, was less important than the attachment to place and social capital associated with aging in place.

## 1. Introduction

Older adults in many parts of the world are aging in rural places. For example, in Australia, over a third of the rural population are older adults [1]; in rural China, there are an estimated 178 million people over 60 years old [2]; and in Sri Lanka, almost 14% of the population is over 60 years old [3]. In Canada, there are approximately 3000 rural communities, 40% of which are not within commuting distance of major hospitals with advanced medical care options. Globally, rural-dwelling adults are a heterogeneous population that includes urban transplants, indigenous peoples, telecommuters, farmers whose families have been on the land for generations, back-to-the landers, and everyone in between. Their rural settings include isolated individual properties, small clusters of homes, villages, and small towns and are all located outside large, densely populated urban centers [4]. Unlike their urban counterparts, rural dwellers generally have a self-reliant existence [5], supplying their own water and sewage systems, and often arranging their own electricity. They are, of necessity, familiar with contingency planning, able to prepare for adverse weather events that shut down roads and power, and are often able to care for themselves and their families for periods of time without assistance.

Globally, rural communities face limited access to transportation, which impacts access to friends, family, and health services [1,6]. The small size of these communities creates challenges in terms of local services available to older adults aging in place. For example, palliative care resources may be limited in rural areas [7,8]. Consistent with the lack of available resources, rural older adults in many regions do not visit health care providers as often as urban residents [1,5,9], and are more likely to go to hospital emergency departments [10] and to be admitted [11]. In general, despite the fact that rural older adults tend to have poorer health [12,13] and less access to health care services [5,14], they express greater satisfaction with their health [15]. The ability to age successfully depends on continued access to social interactions within communities as well as access to health care services. Without these, successfully aging in place in rural communities may be impossible.

Whether urban or rural residents, older adults’ successful aging is often influenced by the ability to participate in social interactions. Social participation is a form of social interaction that includes activities with friends, family, and/or other individuals. It can be one-on-one or in a group. Couton and Gaudet give the examples “helping neighbours, getting involved in informal groups, etc.” [16] (p. 23), and other researchers have used specific categories, such as visiting restaurants or bars, talking on the phone, going to church or doing other religious activities, meeting with friends, attending arts or cultural events, and going to exercise groups [17]. It has been underscored as a priority by human rights organizations due to its role as an important source of meaning and positive experiences [18]. Social participation has also been shown to have health-protective effects in later life [17,19], and participation activities at the community level and within family groups are linked to a sense of belonging, interpersonal social connections [16], and attachment to the geographic location [20] and may be the key to achieving successful aging in this last stage of life [19,21].

### 1.1. Lived Experience and Successful Aging

In order to explore aging in place in such a way that includes the voices of older adults, quotes from research that recorded the way people feel about and understand their choices and actions, known as the lived experience, are incorporated here. We believe that by paying particular attention to lived experience, especially as it applies to the dynamic interaction between the characteristics of the person and the social and physical environments, the appropriate supports to help rural-dwelling adults to age in place can be identified, as can gaps in knowledge and directions for future research.

Lived experience combines the experience of the physical self along with introspection, interaction with others [22], the recognition of changing abilities, and, generally, adapting to them [23]. As Wondolowski and Davis point out, “the meaning of aging is individual and subject to personal interpretation” [24] (p. 262), and for some, aging brings deeper meaning, more spiritual engagement, and a feeling of having a better life after many years of hard work [22]. This is what researchers term “successful aging”, an important construct, because it focuses on assets and abilities and encompasses the process of adjusting to change over the course of one’s life [25]. Contrary to disengagement theory, which suggests that withdrawal from interaction is the natural culmination of aging [26], successful agers usually continue to participate in the community, maintaining social capital through relationships with others and with the community itself [15]. Recognition of physical change, the dichotomy of “what is” versus “what was”, self-reflection, and creativity are also part of the lived experience of successful aging [27]. Continued engagement with life, personal growth, generativity, integrity, and gerotranscendence and reconciliation are all vital to successful completion of the life cycle [28,29]. The goal is to attain wisdom and pursue meaning, and older adults’ lived experience reflects this [17].

Much of the information available on aging in place comes from researchers, health professionals, caregivers, and adult children rather than from those who are actually experiencing aging. Symptoms, (dis)ability, and mobility are measured and recorded, as are personal and medical histories—a process referred to as being “othered” [30]. Othering reduces the client to an object of study. Lived experience, as a research perspective, acknowledges that individuals, not their clinicians or caregivers, are experts on their own lives [31]. The literature that exists on older adults’ lived experience reveals that when aging in place, their needs are often practical and unrelated to health. They want help with repairing and cleaning their homes [32,33,34], traveling to and from activities or appointments [32,33,35], caring for and keeping their animals [36], and interacting with existing friends [32,33].

### 1.2. Aging in Place

Aging in place ideology is promoted worldwide by the World Health Organization (WHO) to avoid the emotional disruption of leaving home and the expense of institutional care [37]. “Place” is not restricted to principal residence, but also includes the community [38]. Aging-in-place initiatives allow older adults to remain at home if they wish to, despite declining resources or functional abilities due to illness, injury, loss of loved ones, and/or loss of income. Those aging in place often have “a strong drive to stay active and to have meaningful social interactions with others, and they also wanted to contribute to the society” [39] (p. 1). Aging in place is cost-effective and therefore generally favored by policy-makers and health service providers [37].

Part of the importance of aging in place is the attachment that many older adults have to the social and physical environment they live in, which in turn contributes to well-being [40,41]. “Throughout our lives the places in which we live reflect aspects of self. And we reflect those places” [42] (p. 21). Despite the acknowledged value of aging in place, approximately one-third of older adults may not be able to do so due to health and financial constraints [43]. If the goal of aging is to do so successfully, three important factors that may predict the degree of success are the ability to age in place, to maintain attachments to social and physical environments, and to overcome the barriers to being able to do so.

Rural adults who remain in their homes and/or communities in their older years can be considered “aging in place.” Some programs and research studies use “aging in place” to refer to older adults living in retirement communities, senior villages, or assisted living facilities [44,45,46,47]. However, the older adults we focus on here are living out their final years aging in place in their own homes, often due to their strong ties with their communities [48,49]. Aging-in-place initiatives are assumed to provide the most desirable options for rural older adults approaching the end of life, since they maintain the individual’s independence, community, and connections with friends, family, and, if applicable, religious/spiritual community [40,44].

Aging in place in a rural community comes with a unique set of challenges for those who design programs and services to help older adults to age in place successfully [1,44,50]. For example, many of the smallest communities do not have health service providers and family physicians [5,15]. These services may be provided periodically, creating issues in terms of service continuity as well as trusting relationships with practitioners. In Canada, some rural hospitals have stopped providing emergency services [51,52] and many lack on-site pharmacies [53]. Commuting distances between clients and low wages impact the recruiting and retention of home care workers [54]. In Canada, volunteer-based community services (for example, nursing care and Meals on Wheels) are not government funded, and many small communities are unable to support them on municipal funds and community donations [15]. In Australia, “most rural areas have little public transport infrastructure so over the life course people come to be dependent on private car use” [5] (p. 58). This means that even when health care services or community supports are available, getting to them may be difficult or impossible.

Given the reduced services in many small rural communities, older adults aging in place are at risk of being imprisoned in their homes by the lack of health care, community, and social services [52]. Many rural older adults believe that their children will help them to “age in place” [15], but ongoing out-migration driven by education, work, or lifestyle opportunities in urban centers greatly reduces the number of people in rural settings available to assist older adults facing challenges [44,55]. However, many of the disadvantages described above are thought to be countered by attachment to community, considered by many to be a reasonable trade-off [44,56]. Additionally, at least in some studies, there is no significant difference in life satisfaction levels reported by urban and rural older adults [56].

Given the importance of social participation in the experience of aging successfully in rural settings, this scoping review was done to explore the factors influencing social participation within a rural community.

## 2. Methods

### Search Strategy

A scoping review of recent literature on aging was performed. Scoping studies “aim to map rapidly the key concepts underpinning a research area and the main sources and types of evidence available” [57] (p. 23). Following the approach outlined by Arksey and O’Malley [57], we (1) identified the research questions, (2) identified the relevant studies, (3) selected the studies, (4) charted the data, and (5) collated, summarized, and reported the results.

Step One: Posing the Research Question

The research question we sought to explore was: What factors enhance, or detract from, the ability to engage in social participation and successful aging in a rural community?

Step Two: Identifying the Research Studies

The Ovid MEDLINE(R) database was used for the search. The database includes research from approximately 4600 international journals in fields such as medicine, nursing, dentistry, veterinary medicine, allied health, and preclinical sciences from 1950 to the present. We also searched using Summon^®^ Service, which, according to ProQuest LLC, is a “discovery service based on a unified index of content. More than 90 content types, 9000 publishers, 100,000 journals and periodicals, and 1 billion records are represented in the index” [58]. Since we were interested in articles written about aging in place in rural communities and social participation and lived experience, we used the search terms “rural”, “lived experience”, AND/OR “social participation” AND “aging” OR “elderly” OR “elder” OR “older” (Table 1). The search was restricted to peer-reviewed English-language articles available online. Potentially relevant articles from the reference lists of identified publications were used if they met the inclusion criteria. All abstracts from the search strategy were reviewed for eligibility.

Inclusion criteria: Participants were older adults; the article was published in the English language between 2004 and 2018; social participation and/or lived experience was the central topic of the paper; the research was concerned with older adults.

Exclusion criteria: Articles that did not discuss rural context, social participation, and/or older adults.

Step Three: Selecting the Studies

**Figure 1:**
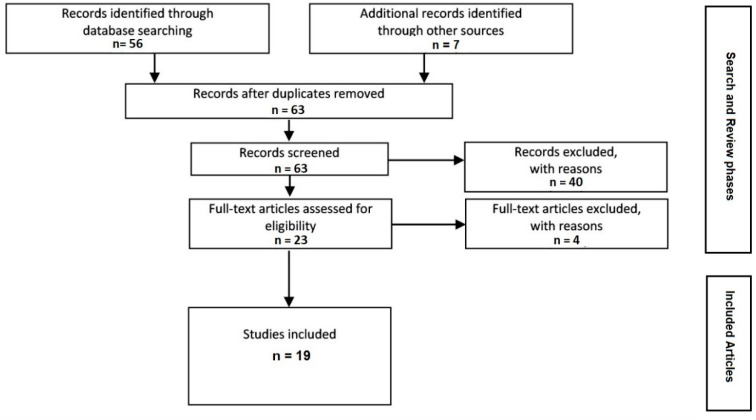
Results of the scoping review.

As shown in Figure 1, the search yielded a total of 63 articles. MEDLINE was the source of 10 articles. The same search terms were put into the Summon search engine, resulting in 46 articles. Another 7 articles were found through reference searches. After the abstracts of the articles were reviewed, 40 were excluded because they did not involve a rural context, social participation, and/or older adults. The full-text review of the remaining 23 articles resulted in the exclusion of a further 4 articles that did not actually discuss social participation, and 19 were retained to be included in the study.

Step Four: Charting the Data

The concepts underpinning the literature on the social participation of older adults aging in rural places included a focus on time spent with others (human and animal), activities with others, and attachment to place (often reflecting “insideness”, the bond between identity and place).

Step Five: Collating, Summarizing, and Reporting the Results

## 3. Results

### 3.1. Being with People and Pets

Social participation includes interactions with others; in this paper we include interactions involving pets as well as those with other people. These interactions can be just spending time with others or doing activities with others, such as volunteering or working. Benefits of social participation include health-protective effects associated with reduced cognitive decline/dementia, better respiration (peak expiratory flow rate), better mental health [19,59,60], successful aging, and quality of life [61]. Bascu, Jeffery, Abonyi, Johnson, Novik, Martz, and Oosman noted, “Participants shared the view that social interaction and keeping active were important factors to support rural healthy aging in place” [62] (p. 334). These Canadian older adults reported that friendship and community activities, including running a business or volunteering, are factors that help healthy aging in their communities [62]. As one participant said, “We feel in a community, you have to be involved. If you’re going to be a recluse, that’s not going to help anybody, including yourself” [62] (p. 332). Park, Kim, and Park found that older Korean women who had higher levels of social participation in community activities expressed higher life satisfaction (b = 0.077; *p* < 0.001) [63]. In Finland, Nummela, Sulander, Rahkonen, Karisto, and Uutela examined social participation among older adults [49]. They found that when comparing urban and rural dwellers, there was more social participation in less populated rural areas, and rural dwellers rated their social capital and self-rated health higher than urban dwellers did [49].

However, not all regions have high levels of social participation among their older adults. For example, Vogelsang found that rural Americans were less likely to engage in social participation activities than their urban counterparts [17]. Similarly, low participation rates were found in rural China [2]. In Australia, Winterton found that a lack of resources, including “limited access to staff, volunteers, buildings, and transport infrastructure; poor-quality physical infrastructure; and limited funding for social initiatives” [64] (p. 265), was linked to a lack of participation in rural activities. Further, isolated older adults were falling through the cracks because no one knew who should be reaching out to them, or even if they could do so while protecting their privacy, as one participant said:

“There’s no doubt that there’s a lot of very disadvantaged, lonely, frail older people in town. And in farm houses around outside of the town … we’re not meeting every older person’s need, not by a long shot”

[64] (p. 270)

Marsh et al. found that, in Sri Lanka, “those most likely to benefit from greater social contact are those most likely to face barriers: older women, the oldest old, the poorest and those in poor health” [3] (p. 13). Barriers existed in China as well, and social participation rates were low [2]. Among 2644 Chinese participants, only 26% engaged in social participation, many of whom were among those with higher education. The researchers hypothesized that the traditional practice of older adults living with younger family members might have played a role in the lower levels of social participation, since “older adults living with others, particularly children or grandchildren, tended to undertake heavy housework and had no time to participate in social activities” [2] (p. 1601). The majority of social participation activities were done through sports or social clubs. Qian et al. suggested that the cultural practice of activities such as tai chi in public settings might play a role in older adults’ interest in sports clubs [2].

The social environment has a huge impact on older adults’ sense of well-being and allows them to maintain an active role in life, to continue growing. This is one older adult’s lived experience: “Every social interaction that I have I feel is always a learning (process). There are so many people who have had experiences that I can glean from. … I can get their knowledge and I can incorporate it into myself and that is growing” [23] (p. 571).

By choosing to live in a rural community, some older adults are actually designing a context for their later life experiences that includes social engagement and community supports in order to continue self-defining roles/activities. Their social networks, built over time, have enabled these individuals to achieve personal goals. One rural-dwelling older adult explained, “Someone stops in just to check on me nearly every day” [32] (p. 85). Another older adult explained that the decision to stay in the rural community was because “that’s where my friends were … the groups I was attending were. Why would I want to move?” [36] (p. 361). Older adults are aware of the importance of their social networks and value the contact with peers and community groups [17,32,36], and because of this some people return to the rural communities of their childhood to retire. As an older adult said, “That’s why we came back [to this town], because we still had friends here and things like that. And it was just like shifting into a nice pair of comfortable shoes! You just carried on! It was just in a different house!” [36] (p. 362). For these older adults, returning to the rural town that they grew up in for retirement is a welcome homecoming [36]. On the other hand, older adults who choose to move to a rural community without previous connections may experience isolation because of their briefer residency [65,66].

Older adults often express the desire to engage in more high-quality [67] social interactions, through recreation or other group activities [68]. In terms of the frequency of social interaction, rural adults may have an advantage over their urban counterparts. Evans looked at older adults in Iowa, USA, and found that the “overall frequency of social interaction among rural residents (M = 2.73, Mdn = 2.00, SD = 2.37) was significantly greater as compared to urban residents (M = 3.01, Mdn = 2.50, SD 2.06), *t* (138) = 2.26, *p* = 0.025” [56] (p. 429). This researcher also found that urban older adults were more likely to feel depressed than their rural counterparts [56]. This depression could be due, at least in part, to survivorship if their older friends and family have died. Vogelsang suggested that social participation might be an element in the creation of meaning and helpful in overcoming grief [17], an issue that is important to many older adults [62].

The ability to be engaged in social interactions, whether with humans or animals, seems to be important to ongoing feelings of well-being, which are in turn linked to the perception of good health and successful aging [41]. Social interactions with pets are becoming more common among older adults [36]. Globally, pets are playing a more and more important role in people’s lives, with many considering them family [69]. Research has documented that the human–animal bond can have a health-protective effect for older adults [70,71], and can facilitate social participation for some people [72,73]. For example, an older adult said, “We’ve got a cat. And I don’t want to part with her. No. She’s part of our lives. She’s not just an animal. She’s part of the family” [36] (p. 363).

Social participation with animals can also support successful aging [71,72]. For example, data from the Canadian Longitudinal Study (CLS), which includes telephone interviews with over 20,000 rural and urban Canadians, showed that “pet ownership was reported by 39 per cent of participants aged 65–69 years, 35 per cent of those aged 70–74 years, 27 per cent of those aged 75–79 years, 22 per cent of participants aged 80–84 years, and 19 per cent of participants 85 years or older” [72] (p. 205). They found that people with pets were not uniformly happier or more satisfied with life than people who did not have pets; however, pet owners who engaged in the most social participation had higher levels of life satisfaction than people who had no pets, but were also engaged in an equivalent level of social participation. Unfortunately, the CLS did not ask about species of pet or record social participation activities that are unique to pet owners, such as dog walking [72] or going to the vet or groomer. These researchers point out that “levels of social participation for the dog owners in our sample, therefore, may have been inadvertently underestimated” [72] (p. 211), as well as the quality of the bond. They also found that many people who are at risk of social exclusion due to race or sexual orientation are also pet owners; in fact, almost 50% of the LGBTQ participants in the CLS were pet owners [72]. Pet ownership itself can be the key, for some people, to overcoming status-based disadvantages [71,72].

Whether with humans or animals, social participation has been demonstrated to be important to ongoing feelings of well-being, which are in turn linked to the perception of good health and successful aging [17,41,62,64]. Given the positive impact that pets can have on social participation [73], Toohey et al. advocate for the integration of pets in age-friendly planning and policies [72].

### 3.2. Doing Activities with and for Others

Rural older adults are more likely than their urban counterparts to develop or continue roles that involve participation in community organizations, including service agencies, local legions, women’s institutes, or religious communities [74,75]. According to the lived experience of an older adult, “I went [to volunteer] thinking that because things turned out so positively for me, I had something to offer them. It turned out the other way around. They were all—every person—had something to offer” [23] (p. 571). Clément, Djilas, Vinet, Aubin, Demers, and Levasseur found that, among Canadian older adults, “volunteering promotes older adults’ involvement in the development of their community and social participation, as well as their physical, mental and social health” [76] (p. 855). The importance of social participation through volunteering has been found by other researchers [76].

Söderhamn, Bjørg, and Söderhamn found that rural-living older adults in Norway were often involved in volunteering with the oldest generation [77]. By helping people older than themselves to shop or go to activities, or visiting with them, it was “possible to care for older people when one’s own parents were gone” [77] (p. 155). These older adults even went so far as to organize evening activities for the older older adults, including dances, which they drove them to and from. Volunteering in this way was a form of social participation that brought meaning and a sense of purpose [77]. However, they also found that the older adults expressed a need for balance between “activity and rest” and “solitude and social interaction” [77] (p. 156). These researchers suggested that the rural environment, which had quite strong social networks, was a factor in these older adults’ ability to remain engaged and continue their social participation.

In Sri Lanka, when they are well enough, older adults often contribute financially and emotionally to their families [3]. They also take the time to engage in religious activities, including participating in funeral rituals and supporting the bereaved. As one Sinhala man in the study said, “Earlier we got married, we produced and raised our children and didn’t have much time for these religious activities, so this is a good time to follow these religious activities in our life” [3] (p. 6). These older adults also engage in volunteer activities, directly benefiting those in the community who need help with meals and medications.

Many rural older adults report a strong sense of belonging in association with membership in community organizations [74], and many rural communities require local older adults to engage in volunteering to augment health services provided by the municipality [78]. It is a win-win in most cases, since volunteering provides meaning for many older adults, allowing them to give back and remain engaged [23]. However, there is a risk that they will become overcommitted due to the limited number of people available to do this work [79].

### 3.3. Attachment to Place

Several articles suggested that community attachment is a fundamental reason for aging in place in a rural location [21,44,80]. If we consider community attachment to be “an individual’s sense (i.e., subjective assessment of) rootedness or fit in the community” [44] (p. 413), then we can understand how identity can be linked to geography. The individual has “become a part of the place to the point where it has become an extension of self” [21] (p. 303). His or her identity is tied to the place that life events unfolded in, creating a sense of insideness that is maintained by trips down memory lane. Much of the research on attachment to place discusses the familiarity of the paths that have been walked for a lifetime, but these linkages of life experience and place are just as important—a drive downtown is a drive down memory lane—and those remembrances reinforce identity, like reviewing one’s personal history, refreshing it at a time of life when memories can be lost.

Social capital can be an important benefit of social participation. Social capital is built through social participation in all aspects of life, including work, school, and hobbies [60]. As a result of being part of a community where they are known and they know people, rural older adults’ social capital has been developed over a lifetime of “instituting acts designed simultaneously to form and inform those who undergo them” [81] (p. 88). Reichstadt, Sengupta, Depp, Palinkas, and Jeste’s qualitative study found that 73% of older adults interviewed reflected on the importance of giving of themselves, maintaining engagement and social capital [17]. Having social interactions and building social capital occur in geographic locations, resulting in a sense of insideness, i.e., the experience of memories of events are linked to place. Insideness can result in attachment to a geographic location (e.g., house, town) that is inextricably linked to identity [21,80]. The individual has “become a part of the place to the point where it has become an extension of self” [21] (p. 303). The older adult’s feeling of insideness is impacted by a lifetime of experiences and is altered by changes in health, mobility, and social networks, all of which bear on the experience of aging in place [82,83].

Many older adults have invested a tremendous amount of time and money in their homes; as a result, they have a bond with that place [32]. An older adult describes the attachment to home: “I lived here for a while when I was young. Bought the place in 1947. It was pretty run down, no running water, no electricity … had to work hard to fix it up” [32] (p. 80). When the environment is familiar and feels safe, the older adult is more likely to want to age in place. As another older adult explained: “We need to stay in our comfort, rather than be uprooted and planted somewhere else, somewhere foreign” [36] (p. 361). However, as one older adult pointed out, a lonely big house is not necessarily comforting, especially if you are the only one to do the cleaning and maintenance: “If you’ve got a big house and your wife dies, there’s only you sat in it, what do you do? You don’t want four bedrooms and two storeys to look after” [36] (p. 360).

If maintaining the home or navigating the environment is too demanding for an older adult, aging in place can become difficult [84], and as a result, some older adults may not wish to stay in the same house as they age. In order to facilitate aging in place in rural communities, older adults report needing help with “housekeeping and home maintenance, transportation, personal care, and management of medical conditions” [85] (p. 780). While including home maintenance in programs for older adults is intuitively a sensible initiative for government programs, this is very rare. Hinck interviewed 19 people, 13 women and 6 men, aged 85 or older living alone in their own homes [85]. Three interviews two weeks apart were conducted in the older adults’ homes. Remaining in their own home was central to the themes of all of the interviews; as one participant said, “Home is a precious place to be, lots of nice memories” [85] (p. 784). Many of these older adults did not express loneliness despite living alone, and most valued the solitude of their rural setting [85]. They were connected to social networks and checked in on each other, without necessarily having to see each other.

Fausset, Kelly, Rogers, and Fisk also interviewed older adults aging at home. Their cohort was 44 individuals aged 66 to 85 years living on their own or with a spouse [86]. Interviews were one-on-one or in a group of up to seven people. Their study was designed to gain a deeper understanding of home maintenance needs of people aging in place. Participants reported difficulty with house- and groundskeeping tasks [86]. Sixteen percent of the tasks these older adults categorized as difficult involved home upkeep, such as heating, ventilation, and air-conditioning maintenance; pest control; light bulb replacement; roof replacement; and maintenance of smoke and carbon dioxide detectors [86].

The interactions between older adults and their environment change with physical and mental health. For example, 80% of frail community-dwelling older adults are unable to participate as much as they wish, often due to mobility constraints [87]. Well-being can be enhanced by adjusting the physical and social environment to facilitate better functioning for older adults (building ramps, installing grab bars, initiating home visitors, increasing contact with social networks). One older adult said, “We need more younger people to read to the housebound, to visit and be neighborly, to maintain the church as a kind of community. Transportation is a big factor for the elderly in this area” [32] (p. 86). Those who can continue to drive will often offer assistance to peers who cannot. As this older adult says: “I can drive, and I can get out. Neighbors call on me to take them shopping and I’m happy to do it for them” [32] (p. 84). In the ever-changing relationship between older adults and their environment, they will stretch themselves in order to continue to interact with both their social and physical environments for as long as possible.

Erickson, Call, and Brown reviewed survey data on 621 older rural residents in rural Utah, USA, and found that scarcity of medical services was a concern for these residents, but in many cases they rated available services highly [44]. “When perceptions of service quality were low, residents were more likely to leave the community to obtain health care” [44] (p. 425). Interestingly, these researchers looked at internet use and found that the longer-term residents of these rural communities were less likely to use the internet. They also found that those who used the internet were less satisfied with their community than those who did not use it. Also, women had a tendency to be more satisfied with their rural communities than men. Overall, Erickson et al. concluded that the older residents of Utah they studied were “content to stay put and age in place with little indication that they are stuck in place” [34] (p. 430).

Life changes, such as moving from the lifetime home to a retirement community, assisted living facility, or other residential arrangement, may cause disruption in social networks and their associated routines and activities as well as dislocate the experience of insideness.

“If I lived anywhere else, I’d be a nobody. I wouldn’t have the position I do.”

Beatrice, 83 years old [21] (p. 303)

An illness- or disability-related move to another community or even to a retirement home in the same community, where the social network is absent, means that the elder has lost a lifetime of investments. It is like asking people to move and leave their financial savings behind. All the resources they have were accumulated in the social network of their community, so not only are they removed from friends, but they lose the resources that they depended on to allow them to successfully navigate life.

Amegbor, Rosenberg, and Kuuire looked at social capital of place rather than of person, finding that rural older adults tended to have “stronger neighborhood social capital and higher neighborhood satisfaction compared to their counterparts living in urban centres” [88] (p. 23), but lower socioeconomic status. These researchers also highlighted the importance of formal and informal support networks for older adults, the presence of which resulted in feelings of greater safety [88].

Bonding social capital, the connection between community members, is often strong among rural older adults, resulting in community strength [2,64], which is important for maintaining attachment to the community, which sustains cohesion. Rural communities may be at as much risk from the in-migration of urbanites looking for idyllic retirement as they are from the out-migration of rural-born younger adults [2].

## 4. Discussion

**Figure 2:**
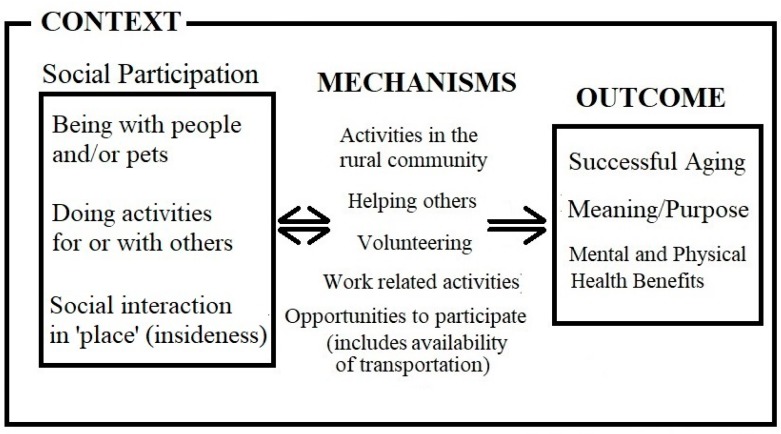
Social participation conceptual framework.

We chose to use a scoping review here because our goal was to quickly capture the key concepts and evidence found in current research on rural aging and social participation, which is precisely what scoping reviews were designed to do [57]. This scoping review of the literature explored social participation among older rural adults aging in place. In the articles we reviewed, social participation was associated with physical and mental health [19,41,59,60,62], and even helped to overcome grief [62]. In response to our research question, as shown in Figure 2, we found that the important factors in positive social participation included being with others, both people and animals; engaging in activities with and for others; and having an attachment to place/community (described as insideness). These social participation activities occur as community-based interactions, formal and informal helping, and work-related interactions, and are supported by services such as transportation to positively influence successful aging [41,61,71,72], meaning/purpose [17,18,23,39,77], and mental and physical health [19,41,59,60,62] in ways that may be bidirectional.

The familiarity of being at home contributes to well-being and successful healthy aging. Despite obstacles, many rural-dwelling elders reported that positive aspects of rural residence, such as continued social participation and the resultant maintenance of social networks within the community, resulted in a sense of belonging that, for many, outweighed the negative (e.g., [21,44,80]). Moreover, by staying in their homes, older adults aging in place within rural communities to which they feel an attachment tend to be able to continue social participation and maintain existing roles and supports. This is important for society, since older adults with these types of resources are more independent of community resources and require less public funding. Aging in place initiatives that support older adults’ independence and ability to remain at home, even in currently underserviced rural communities, are in fact fiscally responsible programs that save taxpayer dollars.

Barriers to social participation included a lack of resources for transportation and social programs [64], and family responsibilities [2]. Remaining in the rural community, especially within the family home, was emphasized as a priority, and help with repairs, cleaning, yardwork, and pet care were often mentioned [33,34,35,36]. Highlighted was that those people least likely to engage in social participation were the most vulnerable: those who had low income or were frail, the oldest old, and those in poor health faced the most barriers to social participation [3].

Multiple studies demonstrate that social participation is enhanced by providing supports for older adults that make it easier to get out of the house and be involved [32,36,85,86]. These supports can be as simple as providing accessible transportation or help with house maintenance or cleaning, which facilitate aging in place, in their homes. Other important supports include ensuring that older adults are not prevented from engaging in volunteer and employment activities.

If we respect the lived experience of older adults, we must listen when they tell us they need to age in place. Based on what older adults are saying so far, the supports required for aging in place are not necessarily health care services, they are human infrastructure—people to help them keep and care for their homes and pets, to drive them, read to them, and check in on them. Interestingly, these all involve outsourcing: getting other people to meet the unmet needs.

### Future Directions

More research is needed to explore the lived experience of aging in place among rural-dwelling older adults. The heterogeneity among rural older adults in these studies, due to the varied geographic areas and the variety in their populations’ characteristics, means that their results can only be generalized to people similar to those surveyed and may not capture the opinions of minority populations. The experiences of older adults who have only recently joined rural communities are also largely unexamined. For recent rural residents, the ability to establish social networks and integrate with the existing rural community could be explored in greater depth by interviewing them to explore their lived experience of the new environment. Much of the research on the health care and end-of-life needs of older adults focuses on urban-dwelling individuals and is guided by researchers. It would be valuable to explore the types of social contact sought by rural-dwelling older adults, the types of environmental impediments they encounter, and their health care needs by listening to the voices of the older adults themselves.

The role of productive and leisure activity in rural older adults’ lives needs to be explored from a lived experience perspective. The leisure activities of long-time rural residents may be missed by researchers who are unfamiliar with traditions of productivity and the resultant activities that seem like they are not recreational. Rural older adults may consider woodworking, quilting, and animal husbandry as leisure activities, whereas nonproductive activities such as golfing and reading may be considered wasteful. In addition, little is known about the importance of animals to the well-being of rural older adults [30]. Many own cats and/or dogs, even farm animals such as chickens, goats, sheep, horses, or cows, or watch wildlife regularly [26]. Perhaps caring for animals, whether taking care of their own pets or feeding wild birds, is a part of remaining productive in later life. Understanding rural older adults’ concepts of productive or meaningful activity is important to any program designed to assist with aging in place.

## 5. Conclusions

Many older adults have planned and prepared for their older years and are, for the most part, healthy [89]. The issue that policy-makers and governments need to consider is not supplying more nursing homes or long-term care beds, but how best to support older adults with aging in place. It is important for local governments to see older adults not as a gray tsunami waiting to engulf them, but as a resource. There are thriving communities “where local leaders see attracting older persons to their communities as a strategy for new economic development and growth” [89] (p. 427). It is important that researchers, policy-makers, and service providers use the lived experience of aging adults to shape decisions. Older adults’ lived experience with respect to aging in place is not just “nice to know”, it is valuable insight that can be used by service providers to guide the process of establishing programs and services.

Some aging-in-place initiatives suggest that remaining in the same community is adequate for older adults, and they do not need to stay in their own homes. However, many older adults express concerns about losing their individuality if relocating to a senior residence is required. For example, some residences designed for elder living have rules that restrict what can be done with gardens, interior and exterior décor, and pet ownership, important aspects of life for many older adults. These constraints limit older adults’ ability to replicate “home” within the residences designed to house them.

This scoping review shows that maintenance of the physical home, assistance with pet care, and transportation to maintain social participation are all vital to increasing older adults’ ability to successfully age in place. Support to do daily tasks (e.g., drive to the grocery store or take the dog to the veterinarian) and have basic human contact (e.g., people reading to them) consists of simple services that allow older adults to age in place successfully, not elaborate retirement residences. With these supports, older adults can continue to contribute to the community through volunteer work and community organizations, which in turn creates a sense of belonging. Many rural-dwelling older adults report that the positive aspects of living in a rural community far outweigh the negative. By being part of a community where they are known and they know people, rural older adults can continue to find meaning and purpose in their final years of life, the key to achieving wisdom in this last stage of the life cycle.

## Figures and Tables

**Table 1:** Search terms used to identify social participation among rural older adults.

Database	Terms
OVID Medline & Summon^®^ Service	^A^ rural and lived experience AND/OR social participation AND aging OR elderly OR elder OR older

^A^ term searched as both subject heading and free text.
